# Capsule commentary on Kurtzman et al., Social incentives and gamification to promote weight loss: the LOSE IT randomized, controlled trial

**DOI:** 10.1007/s11606-018-4603-7

**Published:** 2018-07-27

**Authors:** Samuel G. Smith

**Affiliations:** Leeds Institute of Health Sciences, University of Leeds, Leeds, UK

Kurtzman et al. report a randomized controlled trial testing the effectiveness of gamification interventions on weight loss among 196 adults with obesity.^1^ Participants formed teams with a family member or friend prior to randomization, and were given a goal of 10,000 daily steps and a weight loss goal of 6–8% of baseline weight. Intervention participants also received a multi-component intervention involving weekly targets and entry into a team game. This involved the following: (1) a step count pledge, (2) weekly points linked with the pair’s weigh-in adherence, and (3) a scoring system that promoted or demoted the pair to four levels based on weight loss. One intervention group also had their weight and step data shared regularly with their primary care physician. Financial incentives were provided to participants for completing weigh-ins.

There were within group differences in weight loss at 24 weeks among the intervention groups. However, in keeping with previous trials, ^2^ weight loss also occurred within the control group. There were no significant differences in weight loss between each of the intervention arms and control at 12, 24, or 36 weeks. Exploratory analysis indicated teammates living together achieved more weight loss than those not living together, particularly among the intervention conditions. This finding supports observational evidence, ^3^ and shows promise for the design of future behavioral interventions. Replication in a trial fully-powered for this analysis is warranted.

The intervention was designed by incorporating multiple evidence-based components (e.g., gamification, incentives, targets, etc.), within a single package in the hope of maximizing behavior change. This classical approach is laudable for having the ultimate end-goal in sight: improved patient outcomes. However, when such trials yield null findings we are left with unanswered questions. Did *any* of the intervention components have an effect on the outcome? Were there antagonistic interactions of the intervention components on the outcome? The multiphase optimization strategy (MOST),^4^ suggests such questions should be addressed earlier using an optimization trial, such as a factorial experiment or sequential multiple assignment randomized trial (SMART). When designing behavioral interventions, consideration should be given to using MOST as a guiding framework.
